# Dimethyloxalylglycine Prevents Bone Loss in Ovariectomized C57BL/6J Mice through Enhanced Angiogenesis and Osteogenesis

**DOI:** 10.1371/journal.pone.0112744

**Published:** 2014-11-13

**Authors:** Jia Peng, Zuo Gui Lai, Zhang Lian Fang, Shen Xing, Kang Hui, Chen Hao, Qi Jin, Zhou Qi, Wang Jin Shen, Qian Nian Dong, Zhou Han Bing, Deng Lian Fu

**Affiliations:** 1 Shanghai Institute of Traumatology and Orthopaedics, Shanghai Key Laboratory for Prevention and Treatment of Bone and Joint Diseases with Integrated Chinese-Western Medicine, Ruijin Hospital, Jiao Tong University School of Medicine, Shanghai, China; 2 Department of Orthopaedics, Qian Fo Shan Hospital, Shang Dong University, Ji Nan, China; 3 Department of Orthopaedics, The First Affiliated Hospital of Soochow University, Suzhou, China; University of Alabama at Birmingham, United States of America

## Abstract

Hypoxia-inducible factor 1-α (HIF-1α) plays a critical role in angiogenesis-osteogenesis coupling during bone development and bone regeneration. Previous studies have shown that 17β-estradiol activates the HIF-1α signaling pathway and that mice with conditional activation of the HIF-1α signaling pathway in osteoblasts are protected from ovariectomy (OVX)-induced bone loss. In addition, it has been shown that hypoxia facilitates the osteogenic differentiation of mesenchymal stem cells (MSCs) and modulates Wnt/β-catenin signaling. Therefore, we hypothesized that activation of the HIF-1α signaling pathway by hypoxia-mimicking agents would prevent bone loss due to estrogen deficiency. In this study, we confirmed the effect of dimethyloxalylglycine (DMOG), a hypoxia-mimicking agent, on the HIF-1α signaling pathway and investigated the effect of DMOG on MSC osteogenic differentiation and the Wnt/β-catenin signaling pathway. We then investigated the effect of DMOG treatment on OVX-induced bone loss. Female C57BL/6J mice were divided into sham, OVX, OVX+L-DMOG (5 mg/kg/day), and OVX+H-DMOG (20 mg/kg/day) groups. At sacrifice, static and dynamic bone histomorphometry were performed with micro computed tomography (micro-CT) and undecalcified sections, respectively. Bone strength was assessed with the three-point bending test, and femur vessels were reconstructed and analyzed by micro-CT. Serum vascular endothelial growth factor (VEGF), osteocalcin, and C-terminal telopeptides of collagen type(CTX) were measured by ELISA. Tartrate-resistant acid phosphatase staining was used to assess osteoclast formation. Alterations in the HIF-1α and Wnt/β-catenin signaling pathways in the bone were detected by western blot. Our results showed that DMOG activated the HIF-1α signaling pathway, which further activated the Wnt/β-catenin signaling pathway and enhanced MSC osteogenic differentiation. The micro-CT results showed that DMOG treatment improved trabecular bone density and restored the bone microarchitecture and blood vessels in OVX mice. Bone strength was also partly restored in DMOG-treated OVX mice. Dynamic bone histomorphometric analysis of the femur metaphysic revealed that DMOG increased the mineralizing surface, mineral apposition rate, and bone formation rate. The serum levels of VEGF and osteocalcin were higher in DMOG-treated OVX mice. However, there were no significant differences in serum CTX or in the number of tartrate-resistant acid phosphatase-stained cells between DMOG-treated OVX mice and OVX mice. Western blot results showed that DMOG administration partly rescued the decrease in HIF-1α and β-catenin expression following ovariectomy. Collectively, these results indicate that DMOG prevents bone loss due to ovariectomy in C57BL/6J mice by enhancing angiogenesis and osteogenesis, which are associated with activated HIF-1α and Wnt/β-catenin signaling pathways.

## Introduction

Osteoporosis is a disorder characterized by increased bone fragility, low bone mass, and a consequent increase in fracture risk [1]. Fragility fractures resulting from osteoporosis are the main cause of disablement and death among elderly women. These events lead to the consumption of enormous amounts of medical resources and produce heavy economic burdens [2]. Therefore, using current understanding of disease pathogenesis to develop effective drugs for the prevention and treatment of postmenopausal osteoporosis is vital.

The skeleton is a highly vascularized tissue in which bone remodeling is tightly coupled with angiogenesis. The vasculature supplies nutrients, oxygen, and mesenchymal stem cells (MSCs), which are necessary elements for bone formation, and may direct new bone formation by providing a scaffold for bone-forming cells [3]. Although the main cause of postmenopausal osteoporosis is estrogen deficiency, accumulating evidence from studies in cells, animals, and patients suggests that the local blood supply or decreased angiogenesis contributes to estrogen deficiency-induced osteoporosis [4]–[11].

Angiogenesis depends on hypoxic stimuli and vascular endothelial growth factor (VEGF) production. The hypoxia-inducible factor-α (HIF-α) pathway is the central regulator of the adaptive response to low oxygen levels. The pathway regulates angiogenic genes (e.g.,*VEGF, angiopoietins*). The HIF family comprises three α subunits, HIF-1α, HIF-2α, and HIF-3α. HIF-α is an oxygen-labile protein that forms a heterodimeric complex with the HIF-β subunit, which is constitutively expressed [12]. Under normoxic conditions, HIF-α is hydroxylated at a proline residue by prolyl hydroxylases (PHDs), which need oxygen, iron, and 2-oxyglutarate as cofactors. When hydroxylated, HIF-α is bound by von Hippel-Lindau protein (pVHL), an E3 ubiquitin ligase, and is then degraded by the proteasome. Under hypoxic conditions, prolyl hydroxylation of HIF-α is inhibited, and HIF-α accumulates in the nucleus, where it heterodimerizes with the HIF-β subunit and transactivates HIF responsive genes [13]–[15]. The HIF pathway can be activated under normoxic conditions by small molecule inhibitors of PHDs that interfere with the required PHD cofactors by acting as iron chelators (e.g., desferrioxamine (DFO)) or 2-oxyglutarate analogues (e.g., dimethyloxalylglycine (DMOG)) [14], [16].

Importantly, the HIF-α pathway plays a critical role in angiogenesis-osteogenesis coupling. Clemens *et al*. showed that activation of the HIF-α pathway in mature osteoblasts in developing bone increased bone modeling and the effect was largely attributed to enhanced VEGF-mediated bone vessel formation [17]. Similar results were reported by Giaccia *et al*., who generated mice in which HIF-α was overstabilized in osteoprogenitor cells. The mice exhibited excessive accumulation of trabecular bone in the long bone and increased vascularization [18]. Striking and progressive accumulation of cancellous bone with increased microvascular density and bone formation was also observed in mice lacking *Vhl* in osteochondral progenitor cells [19]. Moreover, the HIF-1α pathway was activated during bone repair and could be manipulated genetically and pharmacologically to accelerate bone regeneration [16], [20]. Consistent with the “promoting osteogenesis by enhancing angiogenesis” theory, HIF-1α pathway activators (DMOG, DFO) or mesenchymal stem cells overexpressing HIF-1α have been shown to improve fracture and bone defect healing [21]–[31].

The HIF-α signaling pathway might be involved in the pathogenesis of estrogen deficiency-induced osteoporosis. Our group reported that bone density, bone vessels, and bone formation were lower in *Hif1a* conditional knockout (KO) ovariectomized(OVX) mice than in wild-type OVX mice [32]. Moreover, the expression of HIF-α and VEGF decreased in OVX mice but not in *Vhl* KO OVX mice. In addition, *Vhl* KO OVX mice, which showed increased angiogenesis and osteogenesis due to activation of the HIF-α pathway, were protected from OVX-induced bone loss [33]. It has been suggested that 17β-estradiol increases HIF-1α and VEGF protein levels and partially stimulates human mesenchymal stem cell (hMSC) proliferation via HIF-1α activation [34]. These data are consistent with the findings of Yen *et al*., who demonstrated that diosgenin, which has estrogenic effects, induces HIF-1 activation and angiogenesis through the src kinase, p38 MAPK, and Akt signaling pathways in osteoblasts [35]. Hence, decreased stimulation of the HIF-1α pathway and angiogenesis might be important factors contributing to estrogen deficiency-induced bone loss. Therefore, activation of the HIF-1α pathway might be a new approach to osteoporosis treatment.

In addition to affecting angiogenesis, hypoxia and hypoxia-mimicking agents increase osteogenesis and modulate Wnt/β-catenin signaling [36]. Hypoxia promotes the proliferation of MSCs and accelerates their differentiation [37], [38]. Similarly, overexpression of HIF-1α in MSCs upregulates the mRNA and protein expression of osteogenic markers *in vitro* [24], [25], [39]. *In vivo* studies using transgenic mice have provided strong evidence that activation of HIF-1α signaling in osteochondral progenitor cells or osteoprogenitor cells increases osteoblast proliferation and differentiation [18], [19]. Treatment of MSCs/osteoblasts with DFO also increases osteogenic marker expression, the effect was attributed to activation of the Wnt/β-catenin signaling pathways [40], [41]. Two different groups have proposed that DMOG not only improves the angiogenic capacity of MSCs, but also enhances their osteogenic differentiation, though the exact mechanism has not been fully elucidated [21], [30]. Considering that reduced osteogenesis of MSCs in postmenopausal women is a causative mechanism for osteoporosis, it is reasonable to promote osteogenesis by activation of the Wnt/β-catenin pathway [42]. Therefore, Wnt/β-catenin pathway stimulation might be a useful approach to osteoporosis treatment [43], [44].

In light of their dual role in angiogenesis and osteogenesis, hypoxia-mimicking agents have tremendous potential for inhibiting bone loss. Supporting this notion, two recent studies have shown that activation of HIF-1α signaling by DFO or inactivation of *Vhl* in osteochondral progenitor cells in a tamoxifen-inducible manner prevented age-induced bone loss [19], [45]. However, iron overload has been regarded as a risk factor for postmenopausal osteoporosis [46], [47]. To exclude the effect of “iron chelation” on the skeleton, we treated OVX mice with DMOG, a cell-permeable prolyl-4-hydroxylase inhibitor that stabilizes HIF-1α under normal oxygen tension by suppressing PHD-mediated HIF-1α degradation [14], [16]. DMOG has shown promise as a therapeutic agent for bone defect healing, neuronal protection, diabetic wound healing, renal protection, and prevention of flap necrosis [21], [30], [48]–[55].

The present study was designed to assess the effect of DMOG on the skeleton of OVX mice by evaluating bone mass, bone microarchitecture, bone biomechanics, and bone turnover. Furthermore, the mechanism by which DMOG alters the HIF-1α and Wnt/β-catenin pathways was investigated.

## Materials and Methods

### Reagents and chemicals

All fine chemicals, including DMOG, tetracycline, dexamethasone, ascorbic acid, β-glycerophosphate, alizarin red S (ARS), 17β-estradiol, l-glutamine, paraformaldehyde, ethylenediaminetetraacetic acid (EDTA), 3-(5′-hydroxymethyl-2′-furyl)-1-benzyl indazole (YC-1),and the tartrate-resistant acid phosphatase staining kit (Leukocyte Acid Phosphatase Assay kit), were purchased from Sigma-Aldrich (St. Louis, MO, USA). Dulbecco’s modified Eagle’s medium (DMEM), fetal bovine serum (FBS), trypsin, penicillin-streptomycin, and TRIzol were purchased from Life Technologies (Gaithersburg, MD, USA). The mouse monoclonal antibodies for HIF-1α and VEGF were purchased from Novus Biologicals (Littleton, CO, USA). The mouse monoclonal antibody for β-catenin was obtained from R&D Systems (Minneapolis, MN, USA), and the mouse monoclonal antibody for β-actin was purchased from Anbo Biotechnology (San Francisco, CA, USA). The rabbit monoclonal antibody for T-cell factor 1 (TCF-1) was from Bioworld Technology, Inc. (St. Louis Park, MN, USA). The lymphoid enhancer-binding factor 1 (LEF-1) polyclonal antibody was obtained from Proteintech Group, Inc. (Chicago, IL, USA). The mouse monoclonal antibody for TATA-binding protein (TBP) was purchased from Abcam (Cambridge, UK). The goat anti-mouse and goat anti-rabbit secondary antibodies conjugated with horseradish peroxidase were purchased from Santa Cruz Biotechnology (Santa Cruz, CA, USA). The VEGF ELISA kit was purchased from R&D Systems (Minneapolis, MN, USA), the osteocalcin ELISA kit was from Biomedical Technologies, Inc. (Ward Hill, MA, USA), and the C-terminal telopeptides of collagen type(CTX) ELISA kit was from Immunodiagnostic Systems (Tyne&Wear, UK). The silicon rubber solution was purchased from Flow Tech (Microfil MV-122; Carver, MA, USA). The primers were synthesized by Invitrogen (Shanghai, China). The RevertAid First Strand cDNA Synthesis Kit was purchased from Thermo Fisher Scientific (Ottawa, Canada). SYBR Premix Ex Taq was purchased from TaKaRa Biotechnology (Dalian, China). The alkaline phosphatase staining (ALP) staining kit was purchased from Shanghai Rainbow Biotechnology (Shanghai, China). The BCA protein assay kit was purchased from Beyotime (Nantong, China). The eECL Western blot kit was obtained from CWBIO (Beijing, China).

### Cell culture and treatment

Murine mesenchymal C3H10T1/2 clone 8 cells were obtained from the American Type Culture Collection (Rockville, MD, USA). The cells were cultured in DMEM supplemented with 10% FBS, 50 U/ml penicillin, 50 mg/ml streptomycin, and 4 mM l-glutamine. Cultures were incubated in a humidified incubator at 37°C and 5% CO_2_. The cells were passaged every 3–4 days. The cells were then plated in 6-well plates at 1×10^5^ cells per well. After 24 hours, the cells were treated with the indicated concentrations of 17β-estradiol, DMOG, or DMOG+YC-1. For the hypoxia experiments, the cells were transferred into humidified incubators at 37°C with 5% CO_2_, and the oxygen tension was reduced to 1% using supplemental N_2_. In order to confirm the effect of DMOG on HIF-1α signaling pathway, the indicated treatment lasted 24 hours. For the experiment about exploration of DMOG on the differentiation of MSCs, the cells were cultured in osteogenic differentiation medium for 7 days. When treatment finished, the cells were collected for western blotting and quantitative real-time PCR analysis. The culture medium was collected for the measurement of VEGF by ELISA.

### Quantitative real-time PCR for mRNA analysis

Total RNA from cells or bone samples were extracted using TRIzol reagent. In order to extract RNA from tibias, the tibias were grounded into pellets after the treatment with liquid nitrogen. cDNA was synthesized using 1 µg of RNA and a RevertAid First Strand cDNA Synthesis Kit. Gene expression was detected with real-time PCR using a SYBR Green qPCR kit and an ABI Step One Plus Real-Time PCR System. The primers used were listed in Table 1.

**Table 1:** Premiers for Real-time PCR analysis.

Gene	Forward Premier	Reverse Premier
VEGF	5′-GGCTCTGAAACCATGAACTTTCT-3′	5′-GCAGTAGCTGCGCTGGTAGAC-3′
β-catenin	5′-GGAAAGCAAGCTCATCATTCT-3′	5′-AGTGCCTGCATCCACCCA-3′
Runx-2	5′-GTGTCACTGCGCTGAAGAGG-3′	5′-GACCAACCGAGTCATTTAAGGC-3′
Osterix	5′-ACCAGGTCCAGGCAACAC-3′	5′-GCAGTCGCAGGTAGAACG-3′
ALP	5′-ACGAGATGCCACCAGAGG-3′	5′-AGTTCAGTVCGGTTCCAG-3′
Osteocalcin	5′-TGCTCACTCTGCTGACCCTG-3′	5′-TTATTGCCCTCCTGCTTG-3′
RANKL	5′-CGTACCTGCGGACTATCTTCA-3′	5′-CTTGGACACCTGGACGCTAA-3′
OPG	5′-CATCGAAAGCACCCTGTA-3′	5′-CACTCAGCCAATTCGGTAT-3′
β-actin	5′-CCCTGTATGCCTCTGGTC-3′	5′-GTCTTTACGGATGTCAACG-3′

### Western blot analysis

After cells or bone samples were lysed, the protein concentrations were measured using a BCA protein assay kit. Each protein sample (40 g) was subjected to SDS electrophoresis and electroblotted onto a polyvinylidene difluoride membrane (0.45 µm; Millipore, Bedford, MA, USA). Afterwards, the membranes were blocked with 5% non-fat dry milk in Tris-buffered saline with Tween 20 (TBST) for 1 hour. The membranes were probed with primary anti-HIF-1α (1∶500), anti-TCF-1 (1∶500), anti-LEF-1 (1∶1000), anti-β-catenin (1∶500), anti-TBP (1∶2000), or anti-β-actin (1∶5000) antibodies at 4°C overnight. The membranes were then washed three times with TBST and incubated for 1 hour with HRP-conjugated secondary antibodies (1∶5000). The antigen-antibody complexes were visualized using the enhanced chemiluminescence detection system as recommended by the manufacturer.

### Alkaline phosphatase staining and alizarin red staining

C3H10T1/2 cells were seeded onto 6-well plates at 1×10^5^ cells per well. After the cells reached confluence, the medium was changed to osteogenesis differentiation medium containing 10^−7 ^M dexamethasone, 0.15 mM ascorbate-2-phosphate, and 2 mM β-glycerophosphate. The cells were cultured for 14 or 21 days and then subjected to ALP or ARS staining. The procedures were conducted according to established protocols. Briefly, the cells were washed with PBS (pH 7.4) three times and fixed with 4% paraformaldehyde (pH 7.4, dissolved in PBS) for 15 min. The cells were then stained with ALP reagent or 0.2% ARS solution for 30 min at 37°C, after which the cells were washed with deionized water three times. The ALP reagent was prepared according to the manufacturer’s instructions.

### Animal models

All animal care and experimental procedures were approved by the Institutional Animal Ethics Committee of Shanghai Ruijin Hospital (ethical approval number: 138). Two-month-old female C57BL/6J mice were obtained from Shanghai SLAC Laboratory Animal Co., Ltd (Shanghai, China). The mice were housed five per cage and were maintained under a strict 12-h light:12-h dark cycle at 22°C with standard food pellets and free access to tap water. After a 2-week adaptation, ten-weeks-old mice were randomly divided into four groups as follows: sham group (ovary intact+vehicle (normal saline) i.p.), OVX group (OVX+vehicle), OVX+L-DMOG group (OVX+5 mg/kg/day DMOG i.p.), and OVX+H-DMOG group (OVX+20 mg/kg/day DMOG i.p.). The ovariectomy was performed in a surgery room exposed to ultraviolet radiation overnight. After anesthetization, the bilateral ovaries were exposed and removed from OVX animals; in the sham animals, the ovaries were exposed but left intact. After surgery, each mouse received an i.p. injection of gentamicin (1000 U) for three successive days. In order to confirm the successful establishment of OVX model, the femoral BMD of 8 mice were measured by *in* *vitro* micro computed tomography (micro-CT) before the surgery, and the BMD of another 8 mice were assessed one month later. The mice were sacrificed by anesthetic overdose. The uterus of each mouse was isolated and weighed. The body weight of each mouse was recorded. The femurs were collected for micro-CT, histological analyses and mechanical test. The left tibia of each mouse was used for PCR analysis, and the right tibia was used for western blot analysis.

### Analysis of micro-CT scans

The distal metaphysic right femur were scanned with a high-resolution (8 µm) micro-CT scanner (GE eXplore Locus SP) to evaluate bone mass, geometry, and trabecular microarchitecture. The parameters computed from these data included bone mineral density (BMD), bone volume/tissue volume (BV/TV), trabecular thickness (Tb.Th), trabecular number (Tb.N), and trabecular separation (Tb.Sp).

### Mechanical testing

The femora from the mice were wrapped in medical gauze saturated with normal saline and stored at 4°C for use the next day. Before testing, the samples were brought to room temperature for 1 hour. The three-point bending test of the left femur was carried out using an Instron 5569 materials testing machine (Instron Inc., Norwood, MA, USA). The femur was placed posterior side down between two supports, which were 6 mm apart. A load was applied at the mid-span, which bent the bone about the anteroposterior axis. Load-displacement curves were recorded at a crosshead speed of 1 mm/s.

### Imaging of femoral blood vessels

Specimens were prepared as previously reported [33]. Briefly, after the mice were euthanized, the thoracic cavity was opened, and the inferior vena cava was dissected and flushed with 0.9% normal saline containing heparin sodium (100 U/ml) to remove the blood from the vessels. Afterwards, the vasculature was perfused with 10% neutral buffered formalin. The vasculature was then injected with 5 ml of silicone rubber compound. Sufficient perfusion was defined as a yellow color change in the lower limbs and liver. The specimens were stored at 4°C overnight, after which the femora were dissected and fixed in 4% paraformaldehyde for an additional 48 hours. The femora were then decalcified in 10% EDTA for four weeks. Images were obtained with a high-resolution (isotropic voxel size of 16 µm) micro-CT imaging system. A threshold of 100 was chosen, and the vessel volume within a region of 5 mm beginning from the distal end of the femur was evaluated.

### Histological preparation and tartrate-resistant acid phosphatase staining

The specimens were decalcified in 10% EDTA for four weeks, and the EDTA solution was changed twice a week. The bones were then dehydrated in a graded series of ethanol washes from 70%–100% before embedding the samples in paraffin. Five-micron-thick longitudinal serial sections were cut and mounted on poly-lysine-coated slides. The deparaffinized slides were washed with PBS three times. The slides were then incubated at 37°C for 60 min in the dark in a solution containing sodium nitrite, fast garnet, naphthol AS-BI phosphoric acid, acetate, and tartrate from the Leukocyte Acid Phosphatase Assay kit, according to the manufacturer’s instructions. Finally, the nuclei were counterstained with methyl green. Multinucleated cells with three or more nuclei were scored as osteoclasts. The average number of osteoclasts per mm of bone surface was then calculated for each femur.

### Fluorochrome labeling and bone histomorphometry

A double tetracycline (25 mg/kg) label was injected subcutaneously at 10 and 3 days before necropsy. At necropsy, the femora were cleaned of soft tissue and fixed in 70% ethanol. The bones were dehydrated and embedded in methyl methacrylate. Thin frontal sections of the femur (5µm) were cut using a microtome (Leica RM2255). Bone histomorphometric parameters were determined according to the report of the American Society of Bone and Mineral Research Nomenclature Committee [56]. Histomorphometric measurements included single-labeled surface (sLS), double-labeled surface (dLS), and interlabel thickness (IrLTh). These data were used to calculate the mineralizing surface/bone surface ratio (MS/BS), mineral apposition rate (MAR), and bone formation rate (BFR) as follows: MS/BS = (1/2sLS+dLS)/BS(%), MAR = Ir.L.Th/Ir.L.t, and BFR/BS = MAR×MS/BS (m^3^/m^2^/day).

### Enzyme-linked immunosorbent assay

Blood from the mice was collected, and the serum was separated by centrifugation at 3000 rpm for 30 min. Serum VEGF, osteocalcin, and CTX levels were measured by ELISA, following the manufacturers’ protocols.

### Statistical analysis

The results were expressed as the mean ± SD. All experimental data were analyzed with one-way analysis of variance (ANOVA) followed by Duncan’s test. *P*<0.05 was considered statistically significant.

## Results

### Effect of 17β-estradiol and DMOG on the HIF-1α signaling pathway

**Figure 1:**
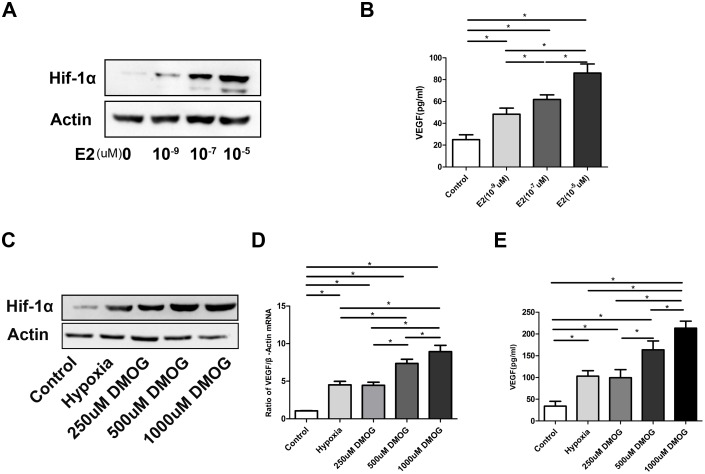
Effect of 17β-estradiol and DMOG on HIF-1α signaling pathway. (A) 17β-estradiol stabilized HIF-1α under normal oxygen pressure. (B) 17β-estradiol upregulated VEGF protein expression. (C) DMOG enhanced HIF-1α protein expression. (D–E) DMOG upregulated VEGF expression at the mRNA and protein levels. *P*<0.05 for comparisons among the groups designated with an asterisk.

Consistent with previous reports, our western blot analysis showed that 17β-estradiol stabilized HIF-1α under normal oxygen tension (Fig. 1A). Furthermore, 17β-estradiol increased the expression of VEGF protein (control vs. 10^−9^E_2_ *P = *0.001<0.05, control vs. 10^−7^E_2_ *P = *0.000<0.05, control vs. 10^−5^E_2_ *P = *0.000<0.05), the main downstream angiogenic target of the HIF-1α pathway, in a dose-dependent manner (Fig. 1B). Similarly, DMOG enhanced HIF-1α protein expression under normal oxygen tension and upregulated VEGF expression at the mRNA and protein levels (Fig. 1C–E).

### Effect of DMOG on MSC osteogenic differentiation and the Wnt/β-catenin signaling pathway

**Figure 2:**
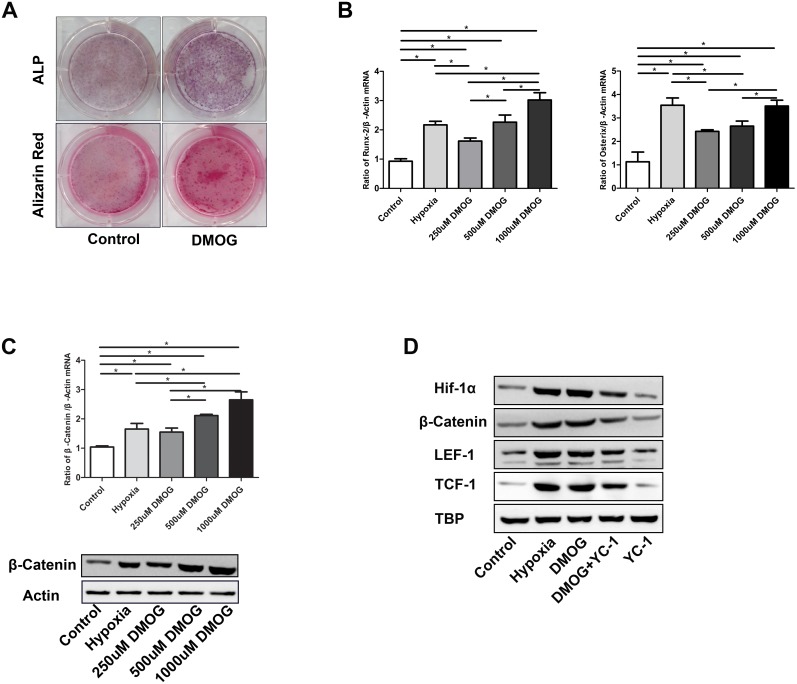
Effect of DMOG on MSC osteogenic differentiation and Wnt/β-catenin signaling. (A) ALP and alizarin red S staining. (B) DMOG promoted RUNX-2 and osterix mRNA expression. (C) DMOG increased β-catenin mRNA and protein expression. (D) DMOG increased nuclear β-catenin, LEF-1, TCF-1, and HIF-1α protein expression. These effects were attenuated by YC-1. *P*<0.05 for comparisons among the groups designated with an asterisk.

ALP and ARS staining showed that DMOG treatment enhanced osteogenic differentiation and calcium deposition (Fig. 2A). Consistent with osteogenesis, RUNX-2 and osterix mRNA levels were increased (RUNX-2 *P = *0.000<0.05, osterix *P = *0.000<0.05) (Fig. 2B). Investigation into the underlying mechanism revealed that DMOG upregulated the levels of β-catenin mRNA and protein (*P = *0.000<0.05) (Fig. 2C). To determine whether the effect of DMOG on Wnt/β-catenin signaling was mediated by HIF-1α signaling, we treated MSCs withYC-1, a widely used HIF-1α inhibitor, along with DMOG and assessed the nuclear expression of β-catenin and the downstream effectors LEF-1 and TCF-1. DMOG stimulated HIF-1α protein expression, and YC-1 inhibited the effect of DMOG on HIF-1α. Furthermore, YC-1 attenuated the DMOG-induced increase in nuclear β-catenin, LEF-1, and TCF-1 protein expression (Fig. 2D).

### Confirmation of the OVX model

OVX increased body weight by approximately 6% when compared to sham treatment (Sham vs. OVX *P = *0.049<0.05). However, DMOG treatment did not affect the OVX-induced body weight gain (OVX+L-DMOG vs. OVX *P* = 0.868>0.05, OVX+H-DMOG vs. OVX *P* = 1.000>0.05) (Fig. S1A). The efficacy of OVX was confirmed by changes in uterine and BMD. The average uterine weights in each group were 0.11137±0.0120 g (sham), 0.0319±0.0079 g (OVX), 0.0337±0.079 g (OVX+L-DMOG), and 0.0326±0.0078 g (OVX+H-DMOG) (*P*<0.0001) (Fig. S1B,C). Before ovariectomy, the BMD of mice was 365.41±18.43 mg/cm^3^, and OVX made the BMD of mice decrease to 281.26±29.61 mg/cm^3^ (*P*<0.0001) (Fig. S1D).

### The effect of DMOG on BMD, bone microarchitecture, and vessels

**Figure 3:**
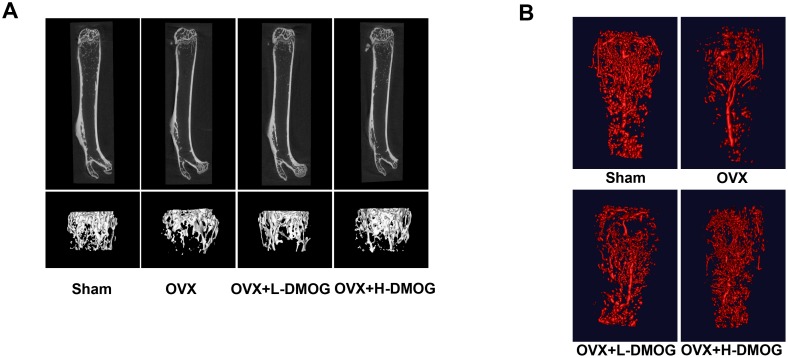
BMD and bone microarchitecture of the trabecular bone in the distal femur. (A) DMOG abrogated the decrease in BMD and the deterioration in bone microarchitecture induced by OVX, as measured by micro-CT. **Morphological analysis of the vasculature within the distal femur from Microfil-perfused mice.** (B) OVX decreased the vessel volume at the distal metaphysic of the femur, while DMOG treatment partly restored vessel volumes.

To characterize the effects of treatment on the trabecular bone compartments, the distal metaphysic of the femoral bone was evaluated with micro-CT imaging (Table. 2). With regard to trabecular bone alterations, OVX mice showed remarkable reductions in BMD, BV/TV, Tb.N, and Tb.Th, as well as increased Tb.Sp (OVX vs. Sham: BMD *P* = 0.000<0.05, BV/TV *P* = 0.000<0.05, Tb.N *P* = 0.000<0.05, Tb.Th *P* = 0.005<0.05, Tb.Sp *P* = 0.000<0.05). DMOG treatment, especially at high doses, improved the trabecular microarchitecture and increased BMD (OVX vs. OVX+L-DMOG: BMD *P* = 0.025<0.05, BV/TV *P* = 0.184>0.05, Tb.N *P* = 0.019<0.05, Tb.Th *P* = 0.94>0.05, Tb.Sp *P* = 0.191>0.05; OVX vs. OVX+H-DMOG: BMD *P* = 0.000<0.05, BV/TV *P* = 0.000<0.05, Tb.N *P* = 0.000<0.05, Tb.Th *P* = 0.047<0.05, Tb.Sp *P* = 0.016<0.05) (Fig. 3A). Microfil-perfused femora were analyzed with micro-CT to assess vascularity. The number of blood vessels in the femur was lower 4 weeks after OVX (*P = *0.000<0.05). However, DMOG treatment led to a dose-dependent increase in femur vessel volume relative to that in the OVX group (OVX vs. OVX+L-DMOG *P* = 0.045<0.05, OVX vs. OVX+H-DMOG *P* = 0.000<0.05), although differences among the DMOG-treated groups and the sham group were evident (Sham vs. OVX+L-DMOG *P* = 0.000<0.05, Sham vs. OVX+H-DMOG *P* = 0.009<0.05) (Fig. 3B).

**Table 2:** Micro-CT analysis of trabecular BMD, bone microarchitecture and vasculature in the distal femur.

	Sham	OVX	OVX+L-DMOG	OVX+H-DMOG
BMD(mg/cm^3^)	379.69±24.61^#^	284.46±17.49*	309.10±19.03*^#^	335.62±30.94*^#^
BV/TV(%)	0.1331±0.0061^#^	0.0947±0.0139*	0.1013±0.0105*	0.1143±0.0119*^#^
Tb.N(1/mm)	3.7268±0.2478^#^	2.5724±0.4024*	2.9317±0.2273*^#^	3.3250±0.3884*^#^
Tb.Th(um)	0.0345±0.0013^#^	0.0322±0.0014*	0.0323±0.0109*	0.0339±0.0025^#^
Tb.Sp(um)	0.2351±0.0211^#^	0.3033±0.0379*	0.2854±0.0183*	0.2693±0.0371*^#^
Vessel volume(mm^3^)	0.4441±0.0396^#^	0.2189±0.0419*	0.2766±0.0243*^#^	0.3529±0.0315*^#^

The groups designated with an asterisk shown significant differences with Sham group (*P<0.05*), the groups designated with a pound sign shown significant differences with OVX group (*P<0.05*).

### Bone mechanical strength examination

The three-point bending test was used to evaluate bone strength (Table. 3). The femurs of the OVX mice exhibited decreases in ultimate stress, ultimate load, energy to failure, and modulus, all of which reflected a decline in bone strength following OVX (Sham vs. OVX: ultimate stress *P = *0.000<0.05, ultimate load *P = *0.000<0.05, energy to failure *P = *0.000<0.05, modulus *P = *0.000<0.05). DMOG treatment improved ultimate stress, ultimate load, energy to failure, and modulus (OVX vs. OVX+L-DMOG: ultimate stress *P = *0.010<0.05, ultimate load *P = *0.005<0.05, energy to failure *P = *0.024<0.05, modulus *P = *0.051>0.05; OVX vs. OVX+H-DMOG ultimate stress *P = *0.000<0.05, ultimate load *P = *0.000<0.05, energy to failure *P = *0.001<0.05, modulus *P = *0.009<0.05).

**Table 3:** Bone quality of the femur as measured by the three-point bending test.

	Sham	OVX	OVX+L-DMOG	OVX+H-DMOG
Ultimate load(N)	15.31±0.85^#^	11.85±0.30*	13.14±0.42*^#^	14.11±0.37*^#^
Ultimate stress(Mpa)	99.41±7.10^#^	69.56±4.88*	80.98±5.59*^#^	90.13±2.37*^#^
Energy to failure(MJ)	8.69±0.50^#^	6.75±0.24*	7.39±0.30*^#^	7.92±0.31*^#^
Modulus(Mpa)	4162.65±377.13^#^	2910.93±355.07*	3407.78±291.51*	3624.83±255.12*^#^

The groups designated with an asterisk shown significant differences with Sham group (*P<0.05*), the groups designated with a pound sign shown significant differences with OVX group (*P<0.05*).

### Serum VEGF, osteocalcin, and CTX levels

The serum level of VEGF, the main angiogenic cytokine upregulated by HIF-1α, was markedly reduced in OVX mice (*P = *0.000<0.05), and DMOG treatment improved VEGF levels in a dose-dependent manner (OVX vs. OVX+L-DMOG *P = *0.003<0.05, OVX vs. OVX+H-DMOG *P = *0.000<0.05). Serum osteocalcin, a marker for bone formation, was higher in OVX mice than in sham mice, but the difference was not significant (*P* = 0.053>0.05). The osteocalcin levels in the DMOG-treated group were significantly higher than those in the OVX group (OVX vs. OVX+L-DMOG *P = *0.008<0.05, OVX vs. OVX+H-DMOG *P = *0.000<0.05). Serum CTX, a marker of bone resorption, was approximately two-fold higher in OVX mice than in sham mice (*P = *0.000<0.05), while DMOG treatment did not affect serum CTX levels (OVX vs. OVX+L-DMOG *P = *0.714>0.05, OVX vs. OVX+H-DMOG *P = *0.770>0.05). All the data were listed in Table 4.

**Table 4:** Biochemical markers of bone in serum as measured by ELISA.

	Sham	OVX	OVX+L-DMOG	OVX+H-DMOG
VEGF(pg/ml)	116.37±6.21#	82.37±8.90*	93.13±5.51*#	100.75±5.31*#
Osteoclacin(ng/ml)	68.33±14.22	80.80±18.87	98.32±9.95*#	107.60±10.91*#
CTX(ng/ml)	16.21±1.61	28.01±1.95*	28.46±4.35*	27.65±2.10*

The groups designated with an asterisk shown significant differences with Sham group (*P<0.05*), the groups designated with a pound sign shown significant differences with OVX group (*P<0.05*).

### Osteoclast number

**Figure 4:**
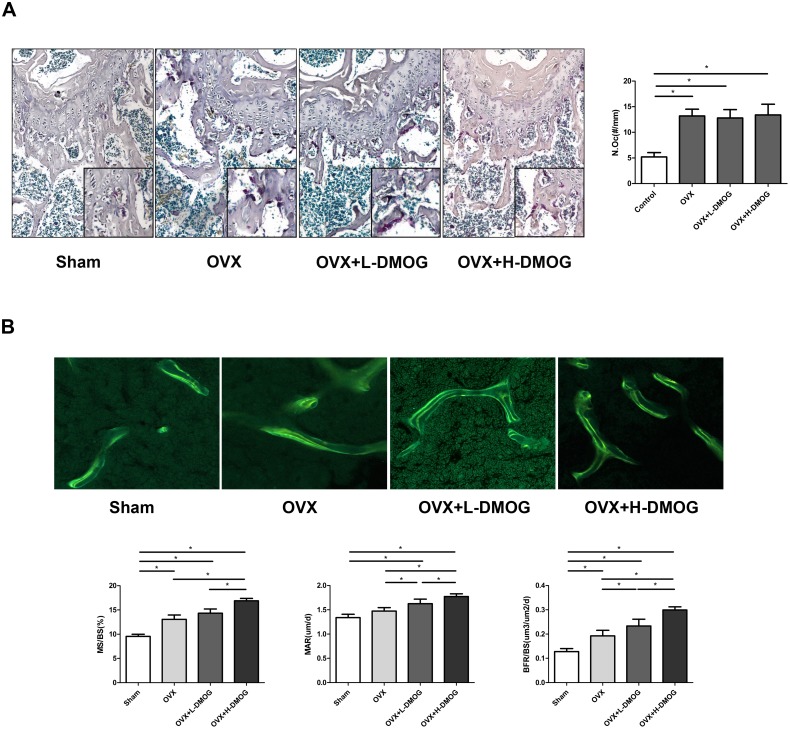
Osteoclast numbers assayed by tartrate-resistant acid phosphatase staining of femoral sections. (A)Osteoclast counting results showed that DMOG treatment had no obvious effect on OVX-enhanced osteoclast formation. Original magnification, ×100. **Dynamic bone formation illustrated by double tetracycline labeling.** (B) DMOG administration promoted bone formation, as evidenced by improved MS/BS, MAR, and BFR/BS. Original magnification, ×200. *P*<0.05 for comparisons among the groups designated with an asterisk.

Consistent with the results of the serum CTX analysis, the TRAP assay revealed a striking increase in osteoclast number following OVX (5.200±0.836 and 13.200±1.303 in the sham group and OVX group, respectively (*P = *0.000<0.05)). DMOG administration did not noticeably prevent OVX-induced osteoclast formation (12.800±1.643 in the OVX+L-DMOG group and 12.600±2.673 in OVX+H-DMOG group; OVX+L-DMOG vs. the OVX group *P = *1.000>0.05, OVX+H-DMOG vs. the OVX group *P = *0.637>0.05) (Fig. 4A).

### Dynamic bone histomorphometry

The dynamic histology of the trabecular bone in the distal femur was compared among the various groups. OVX resulted in significant increases in MS/BS and BFR/BS (Sham vs. OVX: MS/BS *P* = 0.000<0.05, BFR/BS *P* = 0.004<0.05) and increased MAR though without statistical difference (*P* = 0.057>0.05). H-DMOG treatment further increased MS/BS, MAR, and BFR/BS compared with OVX group (MS/BS *P* = 0.000<0.05, MAR *P* = 0.001<0.05, BFR/BS *P* = 0.000<0.05); and L-DMOG administration increased MAR and BFR/BS (MAR *P* = 0.035<0.05, BFR/BS *P* = 0.000<0.05) but not MS/BS (*P* = 0.061>0.05) compared to OVX group (Fig. 4B).

### Alterations in HIF-1α and Wnt/β-catenin signaling in DMOG-treated mice

**Figure 5:**
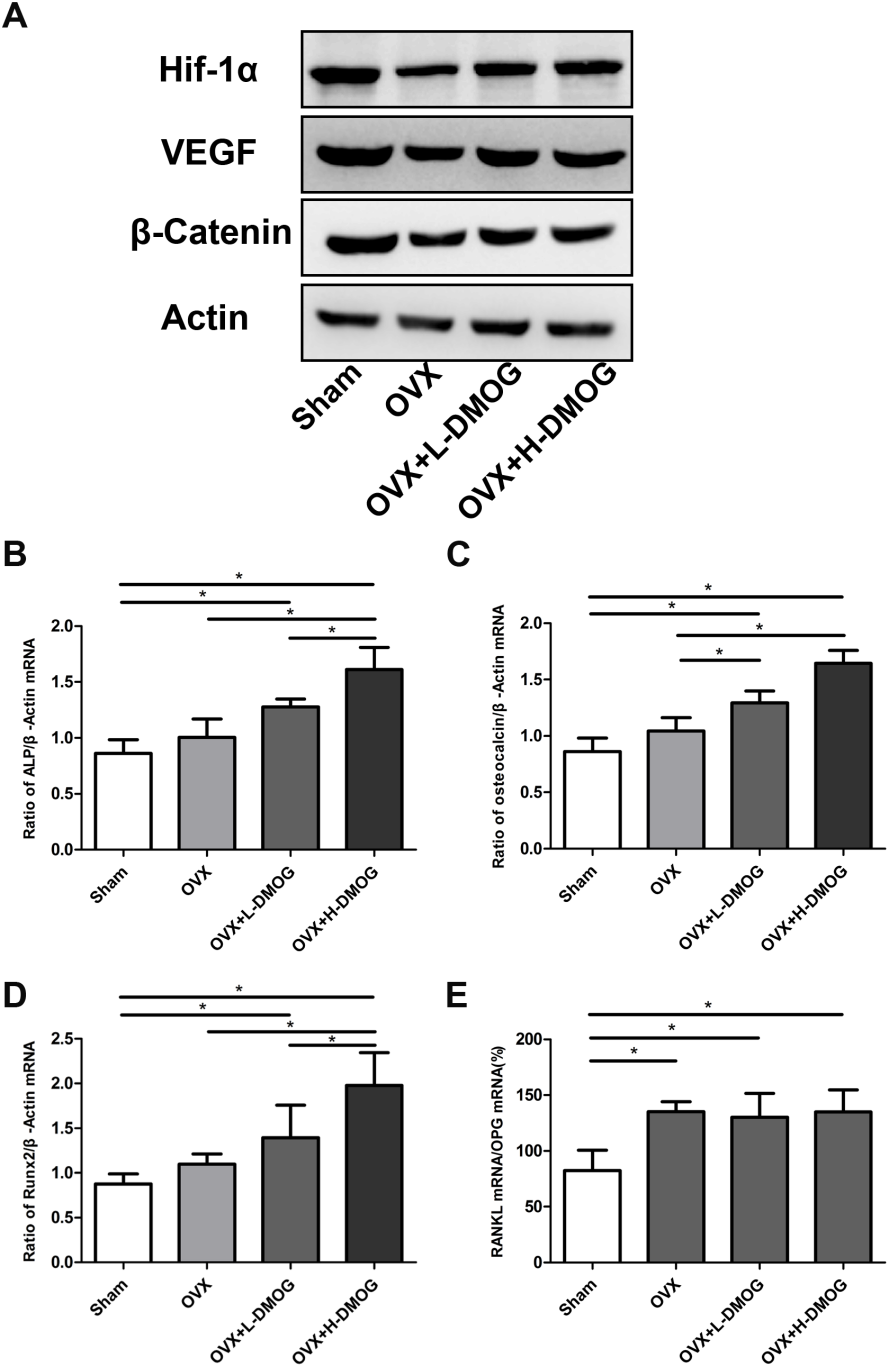
HIF-1α, VEGF, and β-catenin expression in bone samples detected by western blot. (A) HIF-1α, VEGF, and β-catenin expression was lower in OVX mice than in sham mice. However, DMOG treatment increased HIF-1α, VEGF, and β-catenin expression relative to expression in the OVX group. **Effects of DMOG administration on tibial ALP, osteocalcin, RUNX-2, and RANKL/OPG mRNA expression in OVX mice assessed by real-time PCR.** (B) ALP/β-actin ratio. (C) Osteocalcin/β-actin ratio. (D) RUNX-2/β-actin ratio. (E) RANKL/OPG/β-actin ratio. *P*<0.05 for comparisons among the groups designated with an asterisk.

To illustrate the enhanced angiogenesis and osteogenesis in DMOG-treated mice, we used western blot to detect alterations in HIF-1α and Wnt/β-catenin signaling pathways in collected bone samples (Fig. 5A). As previously highlighted, OVX substantially reduced HIF-1α expression; the reduction in HIF-1α was accompanied by a decrease in VEGF expression. However, in the DMOG-treated group, the expression of HIF-1α and VEGF increased significantly. Moreover, DMOG administration enhanced β-catenin expression relative to expression in the OVX group.

### Bone formation and bone resorption markers in DMOG-treated mice

Real-time PCR results for ALP, osteocalcin, RUNX-2, RANKL, OPG, and β-actin expression in each group are shown in Fig. 5B. OVX increased ALP, osteocalcin, and RUNX-2 mRNA levels over those in the sham group, but the difference was not statistically significant (ALP *P = *0.262>0.05, osteocalcin *P = *0.089>0.05, RUNX-2 *P = *0.346>0.05). The osteocalcin mRNA level was higher in the DMOG-treated OVX groups than in the OVX groups (OVX vs. OVX+L-DMOG *P* = 0.030<0.05, OVX vs. OVX+H-DMOG *P* = 0.000<0.05), and ALP and RUNX-2 mRNA levels were markedly higher in the H-DMOG treated group than in the OVX group (ALP *P* = 0.001<0.05, RUNX-2 *P* = 0.004<0.05). In OVX mice, RANKL/OPG expression was markedly increased when compared with expression in the sham group (*P = *0.006<0.05). However, DMOG administration did not significantly attenuate the OVX-induced increase in RANKL/OPG expression (OVX vs. OVX+L-DMOG *P* = 0.733>0.05, OVX vs. OVX+H-DMOG *P* = 0.984>0.05).

## Discussion

In the present study, DMOG improved angiogenesis and osteogenesis *in vitro*. The effects were attributed to activation of the HIF-1α and Wnt/β-catenin signaling pathways, respectively. We investigated whether DMOG ameliorated OVX-induced osteoporosis in mice. Our results showed that DMOG improved BMD, trabecular microarchitecture, and bone strength in OVX mice. In addition, DMOG treatment partly restored the blood vessels of OVX mice. Enhanced osteogenesis might underlie these improvements, as indicated by the acceleration in MS/BS, BFR/BS, and MAR; the increase in serum osteocalcin; and the upregulation of tibial ALP, osteocalcin, and RUNX-2 mRNA. In addition, significant remediation of angiogenesis resulting from increases in VEGF also led to improved bone health in OVX mice.

Stimuli other than hypoxia also cause HIF-1α to accumulate in normoxic cells. For example, growth factors such as insulin-like growth factor can induce HIF-1α synthesis through activation of the PI3 K/Akt/mTOR signal transduction pathway [57], [58]. Here, we found that 17β-estradiol stabilized HIF-1α under normal oxygen tension and increased VEGF protein expression, consistent with previous reports [34], [35]. This phenomenon explains our previous finding that OVX decreased HIF-1α and VEGF expression in osteoblasts [33]. Impaired regulation of HIF-1α is also observed in other pathological processes, such as the development of diabetic wounds, and stabilization of HIF-1α is pivotal for reversing these pathological processes [59]. Mice in which HIF-1α signaling was activated developed extremely dense, heavily vascularized bones, and these mice were protected from OVX or age-induced bone loss [19], [33]. More importantly, age-related bone loss has been prevented by administration of DFO, a hypoxia-mimicking agent [45]. Therefore, stabilization of HIF-1α by a hypoxia-mimicking agent might also help prevent estrogen deficiency-induced bone loss. Another widely used hypoxia-mimicking agent, DMOG, modulated VEGF mRNA and protein levels, which were mainly associated with activated HIF-1α under normal oxygen pressure [14], [16], [20].

In addition to its angiogenic effects, DMOG also has potent osteogenic effects. Enhanced osteogenic differentiation of DMOG-treated MSCs was demonstrated by ALP staining and calcium deposition, which are early and late stage osteogenic markers, respectively. To characterize the underlying molecular mechanism, alterations in the mRNA levels of RUNX-2 and osterix, essential transcription factors for the differentiation of osteoblasts from MSCs [60], were assessed. Our results and those of two recent studies indicate that DMOG upregulates RUNX-2 and osterix mRNA expression [21], [30]. These results were supported by the finding that osteogenesis was enhanced in MSCs expressing a constitutively active form of HIF-1α [24], [25], [39]. Osteoblastic differentiation is predominantly regulated by the Wnt/β-catenin signaling pathway. Wnt binds to Frizzled receptors and their co-receptors, low-density lipoprotein receptor-related proteins (LRP5/6), to stabilize cytosolic β-catenin. β-Catenin then enters the nucleus and stimulates the transcription of Wnt target genes [61]. A reduction in serum β-catenin might contribute to the pathogenesis of postmenopausal osteoporosis [62]. In our studies, DMOG upregulated β-catenin mRNA and protein expression in a dose-dependent manner. Our findings are consistent with those of other groups, which showed that hypoxia and hypoxia mimetics, such as DFO, increase Wnt/β-catenin signaling in MSCs and osteoblasts [40], [41]. As for the relationship between HIF-1α signaling and Wnt/β-catenin signaling, a previous study showed that HIF-1α modulated Wnt/β-catenin signaling in embryonic stem cells [36]. Our studies showed that this phenomenon occurs in MSCs. DMOG increased the expression of nuclear β-catenin and the downstream effectors LEF-1 and TCF-1. The effects were abrogated by the HIF-1α signaling inhibitor YC-1 [63]–[66]. These results suggest that the effect of DMOG on Wnt/β-catenin signaling is mediated by HIF-1α signaling.

In view of the dual role of DMOG in angiogenesis and osteogenesis, we hypothesized that DMOG would attenuate bone loss in OVX mice. As expected, DMOG administration increased the BMD of the trabecular bone. In addition to the BMD, the bone microarchitecture is a critical factor commonly associated with bone quality. Our results showed that BV/TV, Tb.N, Tb.Th, and Tb.Sp, the four fundamental indices of trabecular architecture as determined by the American Society for Bone and Mineral Research, were partly restored to differing extents by DMOG treatment, especially in the H-DMOG-treated group [67]. Because both bone mineral density and microarchitecture, two important factors that determine bone quality, improved significantly, we tested bone strength using the three-point bending test, which showed that DMOG treatment partly rescued the decline in bone mechanical strength due to OVX. These findings strongly suggest that DMOG prevents bone loss and improves bone quality in OVX mice.

Given that angiogenesis and osteogenesis are closely coupled, multiple studies have investigated the role of angiogenesis in the pathogenesis of osteoporosis. Accumulating evidence from studies in cells, animals, and patients indicates that low estrogen levels contribute to fewer vessels or decreases in angiogenic factors, resulting in the progression of osteoporosis [4]–[11]. Our current study supports this idea. OVX decreased the femoral vessel volumes of OVX mice, but femoral vessel volumes were partly restored in the DMOG-treated groups. The alteration in bone vessel volumes was in accordance with that of bone parameters. Furthermore, the restoration of bone vessel volume was coincident with the increased expression of HIF-1α and VEGF in bone samples. VEGF, the best known and most critical angiogenic factor, induces vessel formation and regulates the balance between osteogenic and adipogenic differentiation in MSCs. Mice with VEGF deficiency in osteoblastic precursor cells exhibited an osteoporosis-like phenotype characterized by reduced bone mass and increased bone marrow fat content [68]. Hence, normalization of low VEGF levels in postmenopausal women might be of critical significance.

Compared with antiresorptive drugs, which excessively reduce bone turnover and result in inadequate microdamage repair, drugs targeted towards impaired bone formation might be better suited to the rehabilitation of osteoporotic bone [69]. Our *in vivo* data, including serum osteocalcin, real-time PCR, and dynamic bone histomorphometry results, indicated that DMOG administration further increased osteogenesis in OVX mice [60]. These results are consistent with findings that inactivation of *Vhl* in osteochondral progenitor cells or osteoprogenitor cells increases the proliferation and differentiation of osteoblast lineage cells *in vivo* [18], [19]. Furthermore, the expression of osteoprogenitor and osteoblast markers, including osterix, bone γ-carboxyglutamate protein, and integrin-binding sialoprotein, increased in the long bone of DFO-treated aged mice [45]. The enhanced differentiation of osteoblasts was probably attributed to elevated β-catenin protein expression, which was confirmed by westernblot. In addition to the regulation of the Wnt/β-catenin pathway by HIF-1α signaling, demonstrated *in vitro* in this study, the increased level of VEGF might be another pivotal factor for β-catenin accumulation [70]. Although it has been suggested that activation of the Wnt/β-catenin pathway decreases bone resorption, we did not detect a significant alteration in bone resorption following DMOG treatment, as shown by the serum CTX levels and the osteoclast numbers in the femur [71], [72].

There were several limitations to this study: 1) the BMD and microarchitecture of the lumbar vertebra were not assessed; 2) it would be better if the treatment lasts longer time.

## Conclusions

The results of present study demonstrate that DMOG treatment prevents bone loss, rescues bone microarchitecture deterioration, and restores bone strength in OVX mice. The effects might result from enhanced angiogenesis and osteogenesis. Following DMOG administration, new vessel formation, induced by angiogenic factors, would supply the bone with oxygen, nutrients, and a variety of cells, especially MSCs. Activation of the Wnt/β-catenin signaling pathway would enhance the osteogenic differentiation of MSCs and promote bone formation. To the best of our knowledge, this is the first report to describe the use and dual role of DMOG in the treatment of OVX-induced osteoporosis. DMOG may be a new strategy for the treatment of postmenopausal osteoporosis.

## Supporting Information

Figure S1 **Body weight, uterus weight, representative picture of uterus in each group and BMD alterations before and after OVX.** (A) OVX significantly increased body weight of mice in OVX, OVX+L-DMOG and OVX+H-DMOG groups compared to Sham group. (B) OVX significantly decreased the uterus weight. (C) Representative picture of uterus in each group. (D) OVX obviously decreased the BMD of mice. *P*<0.05 for comparisons among the groups designated with an asterisk.
(TIF)
