# Correction: Dimethyloxalylglycine Prevents Bone Loss in Ovariectomized C57BL/6J Mice through Enhanced Angiogenesis and Osteogenesis

**DOI:** 10.1371/journal.pone.0124702

**Published:** 2015-04-09

**Authors:** The PLOS ONE Staff

In Table 2, the units for Tb.Th and Tb.Sp are incorrect. The units should be in mm. Please see the corrected Table 2 here.

**Table 2:** Micro-CT analysis of trabecular BMD, bone microarchitecture and vasculature in the distal femur.

	Sham	OVX	OVX+L-DMOG	OVX+H-DMOG
BMD(mg/cm3)	379.69±24.61^#^	284.46±17.49*	309.10±19.03*^#^	335.62±30.94*^#^
BV/TV(%)	0.1331±0.0061^#^	0.0947±0.0139*	0.1013±0.0105*	0.1143±0.0119*^#^
Tb.N(1/mm)	3.7268±0.2478^#^	2.5724±0.4024*	2.9317±0.2273*^#^	3.3250±0.3884*^#^
Tb.Th(mm)	0.0345±0.0013^#^	0.0322±0.0014*	0.0323±0.0109*	0.0339±0.0025^#^
Tb.Sp(mm)	0.2351±0.0211^#^	0.3033±0.0379*	0.2854±0.0183*	0.2693±0.0371*^#^
Vessel volume(mm3)	0.4441±0.0396^#^	0.2189±0.0419*	0.2766±0.0243*^#^	0.3529±0.0315*^#^

The groups designated with an asterisk shown significant differences with Sham group (P<0.05), the groups designated with a pound sign shown significant differences with OVX group (P<0.05).
