# Supplementary figures and images for: Dimethyloxalylglycine Prevents Bone Loss in Ovariectomized C57BL/6J Mice through Enhanced Angiogenesis and Osteogenesis
Source: PLoS One. 2014 Nov 13;9(11):e112744. doi: 10.1371/journal.pone.0112744 (PMC4231053; doi:10.1371/journal.pone.0112744)

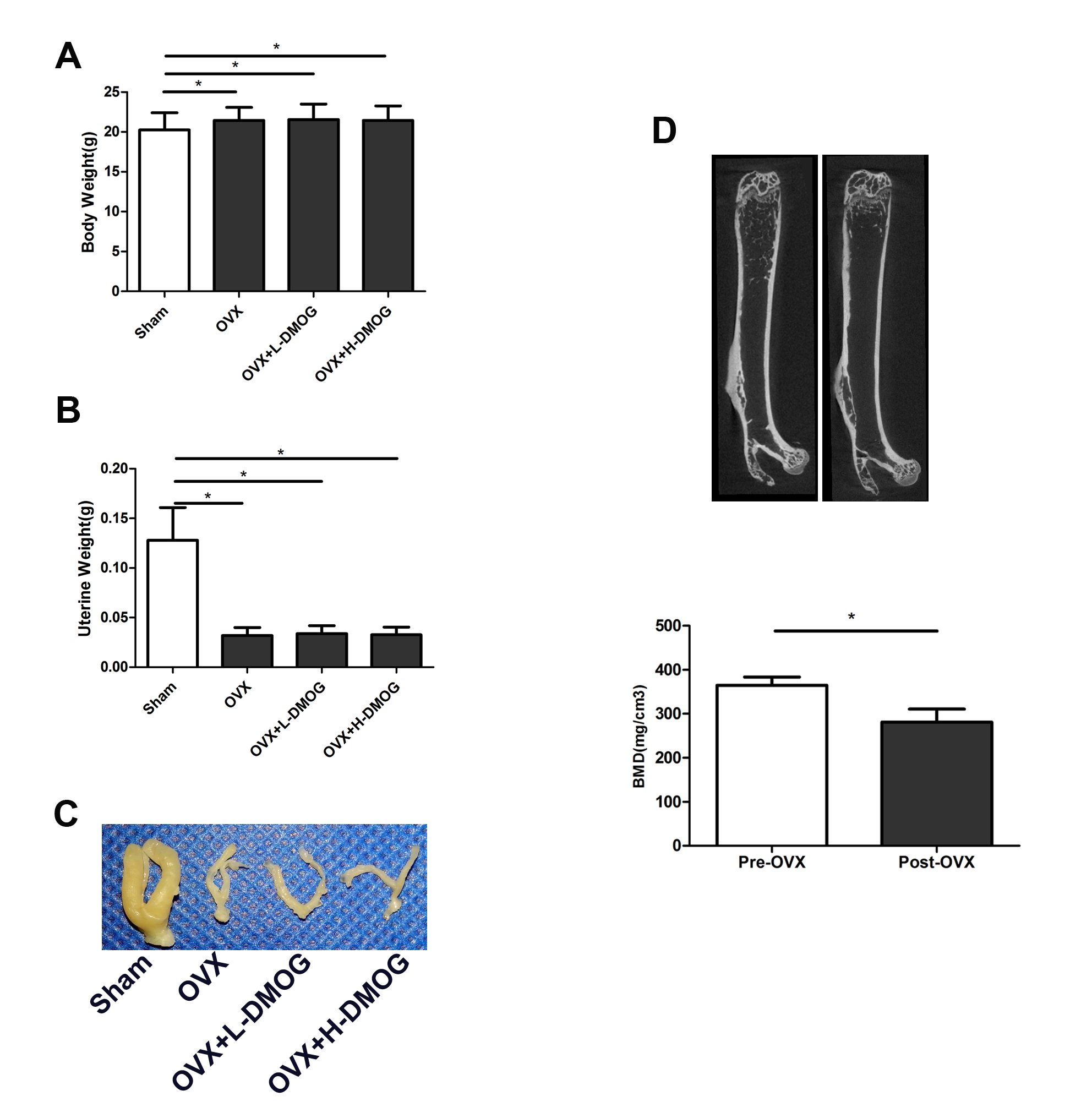

Supplement: Figure S1 — Body weight, uterus weight, representative picture of uterus in each group and BMD alterations before and after OVX. (A) OVX significantly increased body weight of mice in OVX, OVX+L-DMOG and OVX+H-DMOG groups compared to Sham group. (B) OVX significantly decreased the uterus weight. (C) Representative picture of uterus in each group. (D) OVX obviously decreased the BMD of mice. P<0.05 for comparisons among the groups designated with an asterisk. (TIF) [file pone.0112744.s001.tif]
